# Enhancing goals of care discussions: a systematic review and meta-analysis of clinician-directed behavioural nudges

**DOI:** 10.1093/ageing/afag113

**Published:** 2026-04-29

**Authors:** Louisa Poco, Shimoni Shah, Chetna Malhotra

**Affiliations:** Duke-NUS Medical School, Singapore; Lien Centre for Palliative Care, Duke-NUS Medical School, 8 College Road, Singapore 169547, Singapore; Program in Health Services and Systems Research, Duke-NUS Medical School, 8 College Road, Singapore 169547, Singapore

**Keywords:** end-of-life-care, nudges, behavioural interventions, advance care planning, older people, systematic review

## Abstract

**Background:**

Goals of care discussions (GOCDs) are critical for patients with serious illnesses but are hindered by clinician-level barriers. Nudges—low-burden, behaviourally informed strategies that influence clinician behaviour without restricting choice—may provide a scalable approach to increase GOCDs. We systematically reviewed and meta-analysed the effect of clinician-directed nudges on GOCD documentation. Secondary outcomes were quality of communication and healthcare utilisation.

**Methods:**

We searched three databases (2004–2024). Nudges were classified using the MINDSPACE framework. Random-effects meta-analysis estimated pooled odds ratios (ORs) with 95% confidence intervals (CIs) for GOCD documentation. Subgroup analyses examined nudge type, study design and co-interventions. Secondary outcomes were narratively synthesised.

**Results:**

Fifty-one studies were included (16 randomised trials, 7 non-randomised controlled studies and 28 pre-post studies). Salience-based nudges, which make key information more noticeable, were most common (86% of interventions). Most interventions (87%) were multicomponent. Nudges increased GOCD documentation (OR 3.30, 95% CI 2.40–4.53). Multicomponent interventions with a nudge component had larger effects than nudges alone (OR 4.03 vs. 1.89; *P* = .03). No differences were observed by nudge types or study designs. Heterogeneity was high (I^2^ = 96.7%) but leave-one-out sensitivity analyses supported robustness. Nudges improved quality of communication (2/2 studies) and reduced readmissions (2/2), with inconsistent effects on palliative care referrals (5/19) and no impact on hospital length of stay (0/5).

**Conclusion:**

Clinician-directed nudges improve GOCD documentation and represent a scalable, low-cost strategy for quality improvement. Future research should examine a broader range of nudges, multilevel interventions and effects on the quality and timing of GOCDs across settings.

Key Points

- Nudges are effective across study designs and types, with combined strategies producing larger effects.
- Salience-based nudges (e.g. reminders, alerts) are the most commonly tested approaches.
- Nudges may also enhance downstream patient care, including palliative care referrals.
- Clinician-directed nudges improve GOCD documentation and represent a scalable, low-cost strategy for quality improvement.

## Introduction

Discussions between clinicians and patients about treatment goals, based on patients’ values and priorities, are essential for high-quality, patient-centred care [1]. These goals of care discussions (GOCDs) are particularly critical for patients with serious illnesses, helping to guide decisions regarding specific medical treatments [2]. Although clinicians widely acknowledge their importance [3], GOCDs are frequently delayed or do not occur at all, leading to care that is misaligned with patients’ values and goals [4, 5]. For instance, despite national guidelines recommending GOCDs to be incorporated in routine oncological care [6], only a small proportion of patients with cancer receive such communication [7, 8].

Several clinician-level barriers contribute to this gap. Clinicians frequently cite limited communication training [4], fear of distressing patients and competing demands as reasons for not initiating GOCDs [7, 9]. Moreover, the absence of institutional incentives or streamlined workflows can make GOCDs challenging to implement in routine practice [7, 9].

A promising approach to address this is to ‘nudge’ clinicians to facilitate GOCDs [10]. Nudges are low-burden, behaviourally informed strategies that target automatic cognitive processes to alter behaviours, while preserving freedom of choice [11, 12]. When directed towards clinicians in context of GOCDs, nudges aim to lower their cognitive threshold for initiating these conversations. Examples include electronic reminders [13, 14], communication priming tools prior to consultations [15, 16] and peer comparisons [17, 18]. While some studies report that nudges increase GOCDs and documentation [18–21], others find limited or no effect [14, 22], suggesting variability by study design, nudge type and whether nudges are implemented alone or as part of multicomponent interventions. Improving GOCDs may also enhance downstream patient care by improving patient experience and right-siting patients through increased palliative care referrals, reduced hospital stays and decreased use of intensive care.

Prior systematic reviews have demonstrated the effectiveness of clinician-directed nudges in general healthcare settings for improving adherence to evidence-based practices [23, 24]. A recent US-based review of nudges delivered through electronic health records (EHRs) for advance care planning found positive effects on documentation but did not perform a meta-analysis [25]. No global systematic review and meta-analysis has examined the effectiveness of both electronic and non-electronic nudges on GOCDs or whether the impact differs by nudge characteristics or when combined with other interventions.

Thus, our primary aims are to systematically review and meta-analyse the effect of clinician-directed nudges on GOCD documentation, and to examine whether study design or nudge characteristics modify these effects. The secondary aim is to assess whether nudges improve quality of communication and healthcare utilisation outcomes, including palliative care consultations, hospital admissions, hospital length of stay and intensive care unit (ICU) admissions.

## Methods

**Figure 1:**
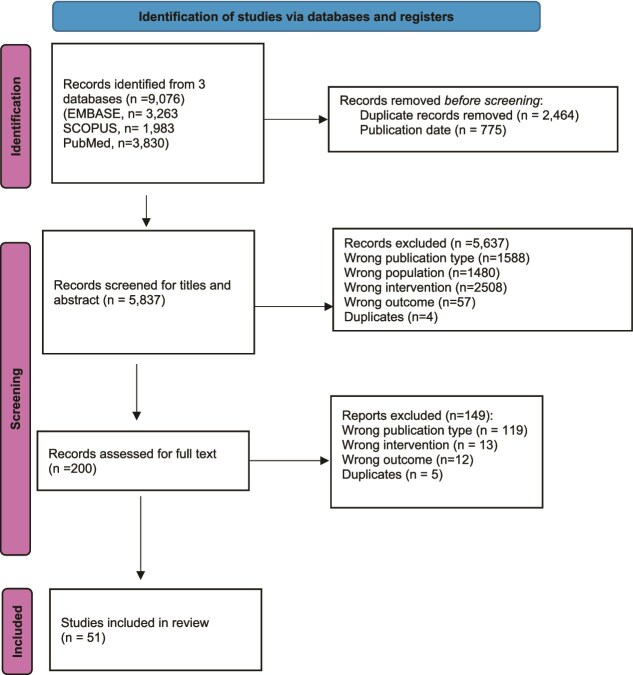
PRISMA flow diagram.

The review was registered in PROSPERO (ID: CRD42025642287) and conducted in accordance with PRISMA guidelines (Figure 1).

### Eligibility criteria

We included quantitative studies evaluating clinician-directed behavioural nudges to encourage GOCDs with patients. Eligible clinicians included physicians, nurses and clinical social workers; lay staff and administrators were excluded. Studies conducted in any healthcare setting were included, and we did not restrict by disease type. No additional eligibility criteria were applied based on patient sample characteristics.

Eligible interventions were those targeting clinicians and containing a behavioural nudge component, either alone or combined with other strategies, to prompt clinicians to initiate GOCDs with patients who may benefit from such discussions. Both electronic (e.g. EHR alerts, e-mail or text reminders) and non-electronic nudges (e.g. in-person prompts, posters) were eligible.

Eligible study designs were Randomized controlled trial (RCT), non-randomised controlled studies with a comparator and uncontrolled pre- and post-intervention studies with baseline outcomes. The primary outcome was GOCD documentation, defined broadly to include serious illness conversations, advance care planning, advance directives, code status and physician/medical orders for life-sustaining treatment. Outcomes could be extracted from electronic or paper medical records or patient self-reports. Studies were required to report quantifiable outcomes for both intervention and comparator groups or pre/post periods. We measured the outcome variable as the proportion of patients who had a GOCD documented. Qualitative studies, case reports, conference abstracts, protocols, clinical guidelines, unpublished studies, reviews, commentaries, non-English language studies and studies published prior to 2004, were excluded, reflecting the recent introduction of behavioural nudges in the literature.

### Search strategy

We conducted systematic searches of PubMed, EMBASE and Scopus (2004—December 2024) using a PICO (Population, Intervention, Comparator, Outcome) framework: clinicians (population), clinician-directed behavioural nudges (intervention), control or pre-intervention groups (comparator) and proportion of patients with GOCD documentation (outcome). Keywords included ‘clinicians,’ ‘healthcare providers,’ ‘behavioural nudges,’ ‘behavioural economics,’ ‘alerts,’ ‘incentives,’ ‘reminders,’ ‘priming,’ ‘goals of care discussions,’ ‘serious illness conversations,’ ‘advance care planning’ and ‘advance directives’ (see Table A1 for full list).

### Study selection

Studies were imported into EndNote, where duplicates were removed. Two reviewers (LRP and SM) independently screened titles, abstracts and full text using the Rayyan software. Conflicts were resolved through discussion or by the third author (CM).

### Data abstraction

One reviewer (LRP) extracted the data from selected studies into an excel sheet and the second reviewer (SS) verified the data. Conflicts were resolved through discussion or by the third author (CM). We extracted: study characteristics (author, year, country, healthcare setting, design, intervention period), patient demographics and diagnosis, clinician type (specialty) and number receiving the nudge, nudge characteristics (mode: electronic versus non-electronic, type classified via MINDSPACE), presence of co-interventions (other clinician, health system or patient interventions) and outcomes assessed. MINDSPACE framework includes nine behavioural principles—**M**essenger, **I**ncentive, **N**orms, **D**efaults, **S**alience, **P**riming, **A**ffect, **C**ommitment and **E**go [11, 12]. Table A2 defines these principles and examples from the included studies [11, 12]. Outcome data included proportions of patients with documented GOCDs and assessment method. For studies reporting multiple effect sizes, we calculated a pooled effect size using a fixed-effects model before inclusion in the meta-analysis.

### Risk of bias assessment

Two reviewers (LRP and SS) independently assessed the risk of bias (RoB). We used RoB2 for RCTs [26], and the Risk of Bias in Non-randomised Studies of Interventions, Version 2 (ROBINS-I V2) for non-randomised and pre- and post-intervention studies.

### Meta-analysis

Effect sizes were calculated as unadjusted odds ratios (ORs). Random-effects meta-analysis was performed on log-transformed ORs, then back-transformed for interpretation. Heterogeneity was assessed using I^2^ and 95% prediction intervals. Leave-one-out sensitivity analyses were conducted to evaluate the influence of extreme values.

### Subgroup analysis

We examined differences by: (i) study design (RCT, non-randomised controlled, pre/post), (ii) nudge type (salience only, salience plus other nudge types, priming/default) and (iii) presence of co-interventions alongside nudges. Between-group differences were tested using the Qb statistic. We also tested whether effect sizes for the subgroups are significantly different from one another, and reported the results between-group Q statistic (Q_b_) and *P*-values [27]. Statistical significance was set at *P* < .05. Analyses were performed in Microsoft Excel and Stata 18.

## Results

The database search yielded 9076 articles of which 5837 unique studies were screened for titles and abstract. After removing duplicates and articles outside the publication date range, a 200 full-text articles were reviewed, and 51 met inclusion criteria (Figure 1).

### Study characteristics

Among the 51 studies, 16 were RCTs, 7 were non-randomised controlled studies and 28 were uncontrolled pre- and post-intervention studies (Table A4). Most studies were conducted in the US (*n* = 47, 92%), with the remainder from Canada (*n* = 2, 4%) and Australia (*n* = 2, 4%). Notably, more than half (55%) of the studies were conducted in the last 5 years (from 2020–2024). About half (*n* = 27, 53%) of the nudge interventions were implemented in the inpatient settings, while about two-fifths were implemented in the outpatient settings (*n* = 21, 41%), and the remaining (*n* = 3, 6%) in both. Only 14 studies (27%) focused predominantly on patients with cancer.

The primary outcomes assessed were documentation of GOCDs, serious illness conversations and advance care planning (*n* = 33, 65%), with the remainder covering end-of-life preferences, advance directives, code status or physician/medical orders for life-sustaining treatment (*n* = 18, 35%). Outcomes were primarily extracted from electronic medical records (EMRs, *n* = 41, 80%) or a combination of EMR and paper medical records or patient surveys (*n* = 7, 14%). One study surveyed patients for the outcomes, while another study extracted outcomes only from PMR, and another study extracted outcomes from a combination of PMR and patient survey.

Twelve of 51 studies did not report patient age. Among the remaining 39 studies, the mean patient age across studies was 70, with reported mean ages ranging from 55 to 87 years. This indicates that included studies predominantly comprised older adults, consistent with the populations most likely to benefit from GOCDs.

### Nudge characteristics (Figure 2)

**Figure 2:**
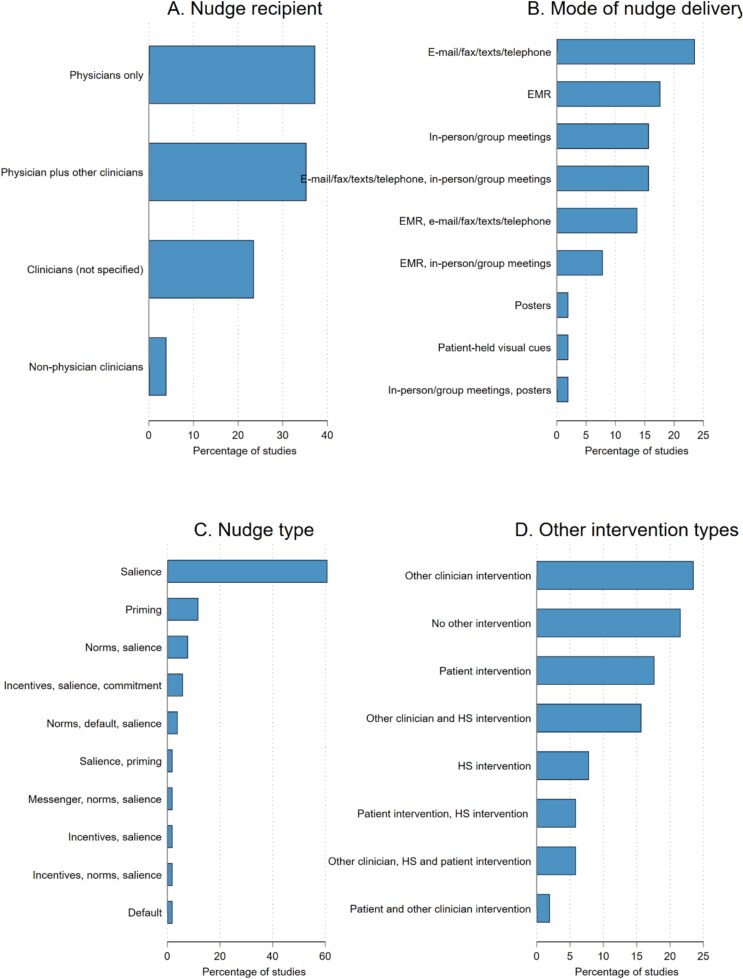
Distribution of nudge characteristics. Abbreviation: HS, health system.

Most interventions targeted physicians exclusively (*n* = 19, 37%), with 18 studies (35%) targeting a mix of physicians, advanced practice clinicians, nurses, medical interns and social workers. Twelve studies (24%) did not specify the clinician type receiving the nudge.

Delivery modes varied: 24% of nudges were delivered via email, fax, text message or telephone; 18% via EMR alerts or notifications; 16% via in-person or group meetings; and 40% used mixed modes. Two studies used only posters [28] or patient-held visual cues [29].

Regarding nudge type, 61% (*n* = 31) were salience nudges, and 25% (*n* = 13) combined salience with other nudge types (messenger, incentives, norms, defaults, priming, commitment). Only six studies [15, 16, 30–33] (12%) used priming nudges exclusively, and one study [34] used default alone. Most studies (78%, *n* = 40) combined nudges with other interventions, including clinician education, health system modifications or patient-directed interventions; 22% (*n* = 11) implemented nudges as a stand-alone strategy.

### Risk of bias

Among RCTs, no study was rated as low risk; 10 (62.5%) had some concerns, and 6 (37.5%) were high risk (Figure A1). For non-randomised controlled and uncontrolled pre-post studies, nearly all had serious or critical risk of bias due to confounding, measurement and selection concerns (Table A3).

### Meta-analysis: association between nudges and GOCDs

**Figure 3:**
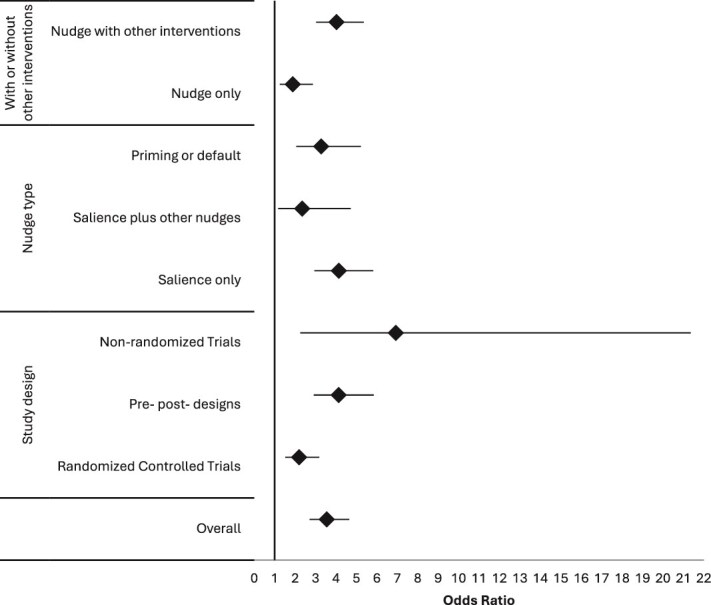
Effects size: overall and by subgroups.

Of the 51 studies, 37 provided sufficient data for meta-analysis. Excluded studies either reported outcomes per admission rather than per patient, lacked sufficient sample sizes or reported outcomes in the long-term. From the meta-analysis, the estimated overall effect size [OR: 4.17 (95% CI: 2.77, 6.26)] is statistically significant. However, the wide prediction confidence intervals (0.37, 46.74) and high I^2^ values (98.07%) indicated substantial heterogeneity among the included studies. Leave-one-out meta-analysis showed that two studies (Lindner *et al.* 2007 [35] and Patel *et al*. 2024 [36]) had a relatively larger influence on the estimation of the effect size. Omitting both studies causes the overall OR to decrease by 20%. To reduce the effect of extreme values in our study, we excluded these two studies. The overall effect size remained significant [OR: 3.30 (95% CI: 2.40, 4.53)] with prediction intervals (0.55, 19.60) and I^2^ of 96.66. We summarised the included studies as a forest plot in Figure 3.

### Subgroup analysis

Effect sizes were consistently positive across study designs: RCTs (OR 2.20; 95% CI 1.52–3.18), non-randomised controlled studies (OR 6.93; 95% CI 2.25–21.36) and uncontrolled pre-post studies (OR 4.13; 95% CI 2.91–5.85), and the differences between groups were not statistically significant (Qb = 3.38, *P* = .18).

All nudge types were effective—salience only (OR 4.14; 95% CI 2.95–5.82), salience plus other nudge types (OR 2.35; 95% CI 1.17–4.72) and priming/defaults (OR 3.28; 95% CI 2.06–5.22)—with no significant difference across types (Qb = 2.06, *P* = .36). Interventions combining nudges with other components showed larger effects (OR 4.03; 95% CI 3.03–5.36) than nudges alone (OR 1.89; 95% CI 1.25–2.87) (Qb = 4.89, *P* = .03).

### Secondary outcomes

Twenty-two of the 51 studies (43%) examined secondary outcomes (Table A5), including palliative care consultations/referrals, quality of communication, hospital admissions and length of stay and ICU admissions. Of the 19 studies assessing palliative care consultations/referrals, 5 [20, 22, 34, 37, 38] (26%) reported a significant increase, 4 using salience-based nudges [20, 22, 37, 38] and 2 [20, 34] implemented the nudge as a stand-alone intervention. Two studies [15, 39] evaluated quality of communication, both showing significant improvement—one used a priming nudge [15] and the other employed a combination of salience and norms nudges [39]. Two studies [28, 40] reported reduced hospital readmissions, both with salience-based nudges combined with other clinician interventions. No study found significant changes in hospital length of stay (salience, 3; default, 1; priming, 1). For ICU outcomes, one study [30] reported reductions in both ICU admissions and length of stay using a priming-based nudge, while another study [21] using a salience-based nudge reported reduced ICU admissions; both were nudge-only interventions.

## Discussion

This systematic review and meta-analysis provides evidence suggesting that clinician-directed nudges increase documentation of GOCDs, though this finding should be interpreted cautiously given the risk of bias observed across many of the included studies. Patients whose clinicians received nudges had 3.3 times higher odds of documented GOCDs compared to those who did not, demonstrating that simple, low-cost, scalable behavioural strategies can meaningfully influence clinician behaviour. However, this estimate may represent an optimistic effect size given methodological limitations across studies. Importantly, this study extends prior work by synthesising evidence for both electronic and non-electronic nudges, conducting a meta-analysis, and examining whether effectiveness differs by nudge type or when combined with other interventions—areas that have not been addressed in previous reviews. From a clinical perspective, these findings suggest that relatively simple changes to clinical workflows, such as reminders, prompts or alerts, may help clinicians initiate GOCDs more consistently, particularly in busy clinical environments where such conversations are often delayed or overlooked.

Our findings are consistent with prior reviews demonstrating the effectiveness of clinician-directed nudges in general healthcare settings for improving adherence to evidence-based practices [23, 24]. They also complement the recent US-based review of EHR nudges for advance care planning, which found positive effects on documentation but did not include a meta-analysis or examine non-electronic nudges [25]. By including both electronic and non-electronic nudges, we provide a more comprehensive assessment of strategies applicable across diverse clinical contexts and healthcare systems. This broader perspective is particularly relevant for geriatric and serious illness care settings, where clinical encounters are complex and opportunities for structured GOCDs may otherwise be missed.

Subgroup analyses showed that nudges were effective across all study designs, nudge types and whether implemented alone or in combination with other interventions. Notably, combined interventions demonstrated larger effects than stand-alone nudges, suggesting that nudges may work synergistically with other strategies such as clinician education, EMR templates or patient-directed interventions. Unlike general healthcare settings where a broad range of nudge types are employed [23, 24, 41], we found salience-based nudges to be most predominant (86%) in our context, reflecting the practical acceptability of reminders and prompts that support clinician awareness without restricting professional autonomy. This may reflect contextual differences in serious illness settings, nudge like defaults or social norms may be less acceptable to clinicians [7], leading to predominance of salience-based nudges—such as reminders and alerts—which support clinician awareness without undermining their professional judgement. Clinically, this suggests that subtle prompts embedded within existing workflows may be more acceptable to clinicians than stronger behavioural interventions that could be perceived as limiting autonomy.

Other nudge types, including priming and default interventions, were also effective, although less frequently studied, highlighting an opportunity for future research to explore the full spectrum of behavioural strategies. Additionally, multicomponent interventions that included a nudge component showed larger effect sizes than interventions with nudges alone. This is unsurprising, as most studies (78%) paired clinician-directed nudges with other clinician, health system and patient interventions, amplifying impact. For instance, salience nudges heightened clinicians’ awareness to conduct GOCDs, but clinician training and EMR templates made conducting and documenting GOCDs easier. These findings suggest that nudges may function best as part of broader implementation strategies that address both awareness and practical barriers to conducting goals of care discussions.

Beyond documentation, secondary outcomes provide preliminary evidence that nudges may positively influence downstream patient care. Increases in palliative care consultations (in 5/19 of the studies) and improvements in communication quality (2/2) were observed, while reductions in hospital readmissions and ICU admissions were reported in a subset of studies. Hospital length of stay, however, was not significantly affected. This pattern suggests that nudges may be most effective for discrete, clinician-controlled decisions that influence palliative care referrals and ICU admissions, whereas broader system-level factors that influence hospital length of stay appear less responsive, likely because they are influenced by multiple factors beyond individual clinician behaviour. Overall, nudges may not only increase GOCD documentation but may also support right-siting of patients and improve patient experience. These findings point to an opportunity to design nudge interventions with improving patient and health system outcomes in mind, as primary endpoints rather than increasing documentation alone.

Despite these strengths, several limitations warrant consideration. First, while nudges improved documentation rates, this does not necessarily imply improvements in the quality of communication. Future studies should evaluate whether nudges lead to high-quality GOCDs. Second, risk of bias was a concern: while RCTs generally showed lower but more precise effect sizes, non-randomised and pre-post studies had larger but less reliable estimates due to confounding and measurement limitations. Third, high heterogeneity across studies reflects differences in patient populations, clinical settings, nudge designs and outcome definitions, which limits the precision of pooled estimates. Fourth, the majority of studies were conducted in North America, restricting generalizability to other healthcare systems. Finally, several studies could not be included in the meta-analysis due to insufficient data, which may limit representativeness.

Given the high risk of bias across studies, future research should prioritize high-quality, multicentre RCTs evaluating both electronic and non-electronic nudges across diverse healthcare systems. Evaluations should extend beyond documentation rates to assess the quality, timing and patient-centredness of GOCDs, and explore mechanisms through which different nudge types exert their effects. Future work should explore optimal timing of nudge delivery as well within the serious illness care trajectory, as prompting clinicians at the clinically meaningful time points may enhance not just GOCD documentation rates but also the quality of conversations and health system outcomes. Multilevel interventions targeting both clinicians and institutional structures, including incentives, defaults and commitment devices [42–46], may provide more sustained improvements in GOCD delivery.

In conclusion, clinician-directed nudges appear to increase GOCD documentation and may influence downstream healthcare utilisation. This systematic review and meta-analysis suggests that low-cost, behaviourally informed strategies have the potential to be integrated into routine clinical practice to support GOCDs. However, given the risk of bias across many included studies, these findings should be interpreted cautiously. Future work should aim to identify the optimal combination of nudges and complementary interventions to improve both the frequency and quality of GOCDs and improve patient outcomes across healthcare systems.

## Supplementary Material

afag113_aa-25-3330-File002

## Research Data Transparency and Availability

All data used for the analyses are in the Supplement.
