# Supplementary material for: Enhancing goals of care discussions: a systematic review and meta-analysis of clinician-directed behavioural nudges
Source: Age Ageing. 2026 Apr 29;55(4):afag113. doi: 10.1093/ageing/afag113 (PMC13125756; doi:10.1093/ageing/afag113)
Supplement: afag113_aa-25-3330-File002 [file afag113_aa-25-3330-file002.docx]

**Appendices**

**Figure A1. ROB-2 Visualization of ROB domains for RCTs included in the study^1^**

**
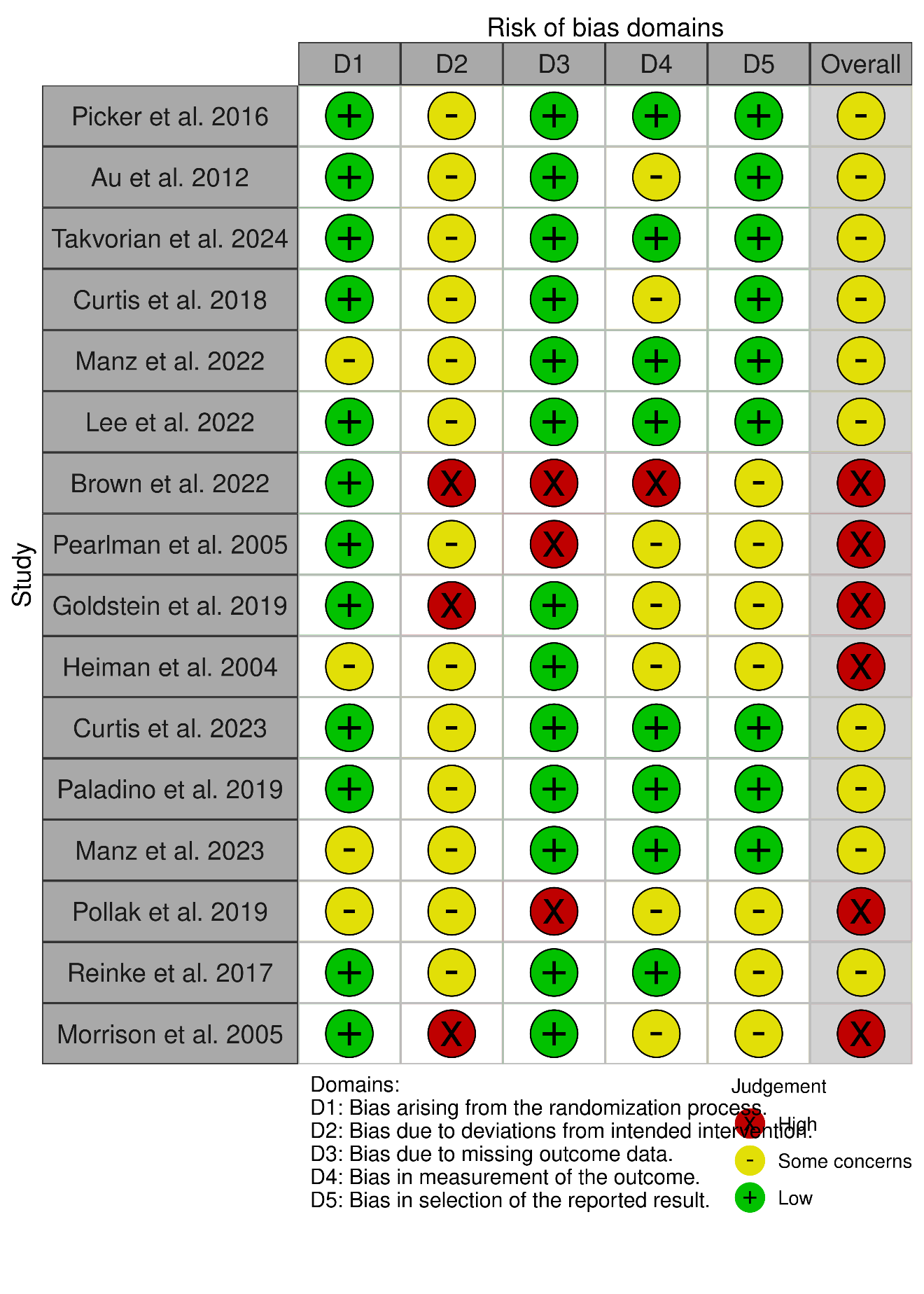
**

**Table A1. Search Strategy**

| **Population** |
| --- |
| "Physicians"[Mesh] OR "Health Personnel"[Mesh] OR "Nurse Clinicians"[Mesh] OR physician[Title/Abstract] OR doctor[Title/Abstract] OR “healthcare professional”[Title/Abstract] OR “health provider”[Title/Abstract] OR “healthcare provider”[Title/Abstract] OR "nurse"[Title/Abstract] OR “primary care clinician*”[Title/Abstract] OR "clinician"[Title/Abstract] OR “outpatient clinic*”[Title/Abstract] OR “hospital setting”[Title/Abstract] OR “general internal medicine”[Title/Abstract] OR “primary palliative care”[Title/Abstract] OR "acute hospital"[Title/Abstract] OR "primary care"[Title/Abstract] OR "electronic medical record"[Title/Abstract] OR "palliative medicine specialist"[Title/Abstract] OR "academic medical center"[Title/Abstract] OR "prognostication score"[Title/Abstract] OR "oncology clinicians"[Title/Abstract] OR “emergency general surgery”[Title/Abstract] OR “palliative care”[Title/Abstract] |
| **Intervention** |
| "Economics, Behavioral"[Mesh] OR “Electronic Health Records”[Mesh] OR “Medical Records Systems, Computerized”[Mesh] OR "Social Comparison"[Mesh] OR “medical record”[Title/Abstract] OR “electronic medical record*”[Title/Abstract] OR “electronic health record”[Title/Abstract] OR behaviour*[Title/Abstract] OR behavior*[Title/Abstract] OR remind*[Title/Abstract] OR performance[Title/Abstract] OR feedback[Title/Abstract] OR “feedback loop”[Title/Abstract] OR nudg*[Title/Abstract] OR “choice architecture”[Title/Abstract] OR “behavioral intervention”[Title/Abstract] OR “behavioural intervention”[Title/Abstract] OR “peer comparison”[Title/Abstract] OR “social comparison”[Title/Abstract] OR “provide information”[Title/Abstract] OR "providing information"[Title/Abstract] OR salience[Title/Abstract] OR “financial incentive”[Title/Abstract] OR incentive*[Title/Abstract] OR “communication-priming”[Title/Abstract] OR “communication priming”[Title/Abstract] OR priming[Title/Abstract] OR “behavioural nudg*”[Title/Abstract] OR “behavioural economics”[Title/Abstract] OR “behavioral economics”[Title/Abstract] OR notifying[Title/Abstract] OR notification[Title/Abstract] OR notify[Title/Abstract] OR notified[Title/Abstract] OR commit[Title/Abstract] OR commitment[Title/Abstract] OR “default option”[Title/Abstract] OR alert*[Title/Abstract] OR chat[Title/Abstract] OR "e-mail"[Title/Abstract] OR "email*"[Title/Abstract] OR “communication strateg*”[Title/Abstract] OR “machine learning”[Title/Abstract] OR “improve communication”[Title/Abstract] OR “communication intervention”[Title/Abstract] OR SICP[Title/Abstract] OR “SICP implementation”[Title/Abstract] OR “serious illness care”[Title/Abstract:~2] OR norm[Title/Abstract] OR norms[Title/Abstract] OR “electronic prompt”[Title/Abstract] OR prompt*[Title/Abstract] OR trigger*[Title/Abstract] OR “personalized message”[Title/Abstract] OR “tailored message”[Title/Abstract] OR “electronic alert”[Title/Abstract:~3] OR “serious illness model”[Title/Abstract:~3] OR "risk prediction"[Title/Abstract:~2] OR “provider interaction with electronic health record”[Title/Abstract:~3] OR "prediction model"[Title/Abstract] OR "computer model"[Title/Abstract] OR "automated notification"[Title/Abstract] OR "patient-specific feedback form"[Title/Abstract] OR "early warning system"[Title/Abstract] OR "early warning"[Title/Abstract] or "warning alert"[Title/Abstract] OR "automated alert"[Title/Abstract] OR “resident-led quality improvement project”[Title/Abstract] OR message[Title/Abstract] |
| **Outcome** |
| "Advance Care Planning"[Mesh] OR “Advance Directives”[Mesh] OR "Patient Care Planning"[Mesh] OR “serious illness conversation*”[Title/Abstract] OR “goals of care discussion”[Title/Abstract] OR “goals of care conversation”[Title/Abstract] OR “goals-of-care discussion”[Title/Abstract] OR “end-of-life discussion”[Title/Abstract] OR “end of life conversation”[Title/Abstract] OR “advance care planning”[Title/Abstract] OR “end-of-life document*”[Title/Abstract] OR “goals of care document*”[Title/Abstract] OR “goals of care discuss*”[Title/Abstract] OR “documented discussion”[Title/Abstract] OR “documented conversation”[Title/Abstract] OR “ACP document”[Title/Abstract] OR “SIC document”[Title/Abstract] OR GOCD[Title/Abstract] OR “documented GOCD”[Title/Abstract] OR “GOC document*”[Title/Abstract] OR “documented ACP”[Title/Abstract] or “documented AD”[Title/Abstract] OR “documented SIC”[Title/Abstract] OR “care review”[Title/Abstract] OR “code status document*”[Title/Abstract] OR “advance directive”[Title/Abstract:~3] OR “care goals”[Title/Abstract] OR “quality of conversation”[Title/Abstract:~3] OR “discussions end-of-life care”[Title/Abstract:~1] OR "GoCC"[Title/Abstract] OR "goals of care"[Title/Abstract:~2] OR "end-of-life communication"[Title/Abstract] OR "advance directive documentation"[Title/Abstract] OR "SIC documentation"[Title/Abstract] OR “advanced directive”[Title/Abstract] |

**Table A2. MINDSPACE framework***

| **Type of nudge** | **Definition** | **Example in the context of goals of care (GOC) discussions** |
| --- | --- | --- |
| ***Messenger*** | The weight we give to information depends greatly on the reactions we have to the source of that information or the "messenger" We are affected by the perceived authority of the messenger, or those we consider credible or trustworthy. | Experienced/ head physician or junior staff member as a messenger of information. The former is a better messenger of GOC-related information than the latter as considered more credible. |
| ***Incentives*** | Incentives such as rewards and punishments are used as a mechanism to motivate behaviour change. Our responses to incentives are shaped by predictable mental shortcuts such as strongly avoiding losses. | Financial incentives can be used to incentivize clinicians to conduct GOC discussions with patients. |
| ***Norms*** | Social and cultural norms are the behavioural expectations, or rules, within a society or group. Norms can be explicitly stated or implicit in observed behaviour. Some social norms have a powerful automatic effect on behaviour and can influence actions in positive and negative ways. This power comes from the social penalties for non-compliance, or the social benefit that comes from conforming | Highlighting the behavior of others (e.g., describe the GOC rates of others) or highlighting what others ought to do will make clinicians want to conform to social norms. |
| ***Default*** | Defaults are the options that are pre-selected if an individual does not make an active choice. Defaults exert influence as individuals regularly accept whatever the default setting is, even if it has significant consequences. We go with the flow with pre-set options. | The default of automatically having an ACP for specific types of patients (high risk) and clinicians will have to explicitly "opt out" |
| ***Salience*** | Our behaviour is greatly influenced by what our attention is drawn to. People are more likely to register stimuli that are novel, accessible, and simple. Simplicity is important because our attention is much more likely to be drawn to things that we can easily understand. | Stimuli such as reminders, alerts in the EMR, e-mails/texts targeted to clinicians. These salience nudges make information more noticeable and increases the likelihood that the clinician will conduct GOC discussions. |
| ***Priming*** | Priming (or activation of any sort) of knowledge in memory makes it more accessible and therefore more influential in processing new stimuli. People behave differently if they have been ‘primed’ by certain cues beforehand | Use information obtained from patients with serious illness about their preferences for GOC communication and send to clinicians as priming tool to support GOC communication between patients and clinicians. |
| ***Affect*** | Affect (the act of experiencing emotion) is a powerful force in decision-making. Emotional responses to words, images and events can be rapid and automatic, so that people can experience a behavioural reaction, and use emotional evaluations as the basis of decisions, before they realise what they are reacting to and before cognitive evaluation takes place | Using emotionally charged strategies or messages to facilitate GOC discussions |
| ***Commitment*** | The conscious act of pre-commitment to a behaviour may subconsciously improve ensuing behaviours, as people strive to deliver on public commitments | Clinicians to promise to the hospital administrators that they will increase the GOC discussion rates as this is one of the goals of the hospital. This commitment will make them want to conduct more GOC discussions |
| ***Ego*** | We tend to behave in a way that supports the impression of a positive and consistent self-image. Our desire for positive self-image leads to an (often automatic) tendency to compare ourselves against others and ‘self-evaluate’. When we make these comparisons, we are biased to believe that we perform better than the average person in various ways. | Emphasize the positive aspects of being able to do their jobs well. Framing the act of conducting GOC discussions positively. |

***Adapted from Dolan et al. (2012)**

**Table A3. Risk of Bias Assessment for Non-Randomized Studies using ROBINS-I V2**

| **Author** | **ROB** | **Study type** |
| --- | --- | --- |
| Chi et al. 2023^2^ | Serious | Non-randomized controlled studies |
| Courtright et al. 2019^3^ | Critical | Non-randomized controlled studies |
| Gensheimer et al. 2022^4^ | Critical | Non-randomized controlled studies |
| Levy et al. 2024^5^ | Serious | Non-randomized controlled studies |
| Lakin et al. 2017^6^ | Critical | Non-randomized controlled studies |
| Wissow et al. 2004^7^ | Critical | Non-randomized controlled studies |
| Ma, C et al. 2020^8^ | Critical | Non-randomized controlled studies |
| Bhattacharya et al. 2023^9^ | Critical | Uncontrolled pre- and post-studies |
| Casarett et al. 2022^10^ | Critical | Uncontrolled pre- and post-studies |
| Colley et al. 2023^11^ | Critical | Uncontrolled pre- and post-studies |
| Conduit et al. 2021^12^ | Critical | Uncontrolled pre- and post-studies |
| Deptola and Riggs 2019^13^ | Critical | Uncontrolled pre- and post-studies |
| Dutta et al. 2024^14^ | Critical | Uncontrolled pre- and post-studies |
| Fang et al. 2022^15^ | Critical | Uncontrolled pre- and post-studies |
| Haley et al. 2017^16^ | Critical | Uncontrolled pre- and post-studies |
| Halpert et al. 2022^17^ | Critical | Uncontrolled pre- and post-studies |
| Hanson et al. 2005^18^ | Critical | Uncontrolled pre- and post-studies |
| Hanson et al. 2017^19^ | Critical | Uncontrolled pre- and post-studies |
| Hayek et al. 2014^20^ | Critical | Uncontrolled pre- and post-studies |
| Haynes et al. 2019^21^ | Critical | Uncontrolled pre- and post-studies |
| Horecki et al. 2024^22^ | Critical | Uncontrolled pre- and post-studies |
| Kantor et al. 2021^23^ | Critical | Uncontrolled pre- and post-studies |
| Karim et al. 2018^24^ | Critical | Uncontrolled pre- and post-studies |
| Lindner et al. 2007^25^ | Critical | Uncontrolled pre- and post-studies |
| Mathew et al. 2023^26^ | Critical | Uncontrolled pre- and post-studies |
| Oppenheim et al. 2022^27^ | Critical | Uncontrolled pre- and post-studies |
| Patel et al. 2024^28^ | Critical | Uncontrolled pre- and post-studies |
| Reed et al. 2020^29^ | Critical | Uncontrolled pre- and post-studies |
| Schell et al. 2023^30^ | Critical | Uncontrolled pre- and post-studies |
| Serna et al. 2024^31^ | Critical | Uncontrolled pre- and post-studies |
| Sorge et al. 2024^32^ | Critical | Uncontrolled pre- and post-studies |
| Switzer et al. 2019^33^ | Critical | Uncontrolled pre- and post-studies |
| Temel et al. 2013^34^ | Critical | Uncontrolled pre- and post-studies |
| Topoll et al. 2022^35^ | Critical | Uncontrolled pre- and post-studies |
| Wasp et al. 2022^36^ | Critical | Uncontrolled pre- and post-studies |

**Table A4. Study characteristics**

|  | **Author/ Year/ Country** | **Study design** | **Setting/ Cancer disease group** | **Mode of nudge delivery** | **Nudge recipient** | **Nudge intervention** | **Nudge type** | **Other interventions incorporated** | **Documentation outcome / source of record/ level** | **Outcome for pre- / control group** | **Outcome for post- / intervention group** |
| --- | --- | --- | --- | --- | --- | --- | --- | --- | --- | --- | --- |
| 1 | Au et al. 2012, US^37^ | RCT | OP, No | In-person/group meetings | Physician plus other clinicians | 1-page patient-specific feedback form | Priming | Patient intervention | End-of-life preferences discussion, patient survey, patients | N = 182, 15.9% | N= 194, 35.2% |
| 2 | Bhattacharya et al. 2023, US^9^ | Uncontrolled pre- and post- study | IP, No | Posters/stickers | Physicians only | Reminder visual cues on eligibility criteria were posted at workstations if patients met any criteria. | Salience | Other clinician intervention | MOLST completion, EMR, patients | N = 149, 1.3% | N = 108, 11.1% |
| 3 | Brown et al. 2022, Australia^38^ | RCT | IP, No | EMR, e-mail/fax/texts/telephone | Clinicians (not specified) | Notification alert attached to patient EMR/ medical report or visual flag displayed on the electronic patient journey board. E-mail notifications were also sent. | Salience | No other intervention | Review of care directive orders, EMR and PMR, patients | Hospital X: N=521, 21%, Hospital Y: N=1304, 19%, Hospital Z: N=310, 23% | Hospital X: N=1317, 11%, Hospital Y: N=480, 24%, Hospital Z: N=298, 23% |
| 4 | Casarett et al. 2022, US^10^ | Uncontrolled pre- and post- study | IP and OP, No | EMR | Clinicians (not specified) | Best practice advisory notification on a patient's home screen if no GOCD has been documented. | Salience | Other clinician, HS and patient intervention | GOCD, EMR, aggregated monthly | N= not reported, 2016: 3% | N = not reported, First half of 2022: 50% |
| 5 | Chi et al. 2023, US^2^ | Non-randomized control study | IP, No | EMR | Physicians only | Notify via EMR chat alerting them of the patient’s elevated risk score, messages encouraging SICs. | Salience | No other intervention | GOCD, EMR, patients | N = 168, 16% | N=168, 60% |
| 6 | Colley et al. 2023, US^11^ | Uncontrolled pre- and post- study | IP, No | E-mail/fax/texts/telephone | Physicians only | E-mail to review ACP process and encourage ACP documentation. | Salience | Other clinician and HS intervention | ACP, EMR, patients | N=590, 29% | N=555, 32% |
| 7 | Conduit et al. 2020, Australia^12^ | Uncontrolled pre- and post- study | OP, Yes | In-person/group meetings | Physicians only | Status of patients’ GOC was identified and verbally communicated to the physicians. | Priming | Other clinician intervention | GOCD, EMR, not reported | Not reported | Not reported |
| 8 | Courtright et al. 2019, US^3^ | Non-randomized control study | IP, No | E-mail/fax/texts/telephone | Clinicians (not specified) | Nurses offered triggered palliative care consults to physicians until two consults were accepted, physicians can opt out. | Default | No other intervention | ACP, EMR, patient admissions | N = 138, 17% | N=134, 28% |
| 9 | Curtis et al. 2018, US^39^ | RCT | OP, No | E-mail/fax/texts/telephone | Physician plus other clinicians | 1-page patients specific feedback form | Priming | Patient intervention | GOCD, EMR and patient survey, patients | N =211, 31% | N=175, 74% |
| 10 | Curtis et al. 2023, US^40^ | RCT | IP, No | E-mail/fax/texts/telephone | Physician plus other clinicians | 1-page patient-specific feedback form, intervention e-mail reminder | Salience, priming | No other intervention | GOCD, EMR, patients | N=1257, 30.4% | N=1255, 34.5% |
| 11 | Deptola and Riggs, 2019, US^13^ | Uncontrolled pre- and post- study | IP, No | E-mail/fax/texts/telephone | Physicians only | E-mail alert with information on patient’s mortality risk | Salience | No other intervention | GOCD, EMR, patients | N = 151, 49% | N=132, 69% |
| 12 | Dutta et al. 2024, US^14^ | Uncontrolled pre- and post- study | IP, No | EMR, in-person/group meetings | Clinicians (not specified) | ACP documentation rate was distributed twice monthly | Incentives, salience, commitment | Other clinician and HS intervention | ACP, EMR, patients | N=4306, 14% | N=5914, 44% |
| 13 | Fang et al. 2022, US^15^ | Uncontrolled pre- and post- study | OP, No | E-mail/fax/texts/telephone, in-person/group meetings | Physician plus other clinicians | Written notification and an e-mail reminder of high-risk patients | Salience | Other clinician and HS intervention | GOCD, EMR, patients | N=47, 0% | N=40, 88% |
| 14 | Gensheimer et al. 2022, US^4^ | Non-randomized control study | OP, Yes | EMR, e-mail/fax/texts/telephone | Physicians only | Weekly e-mails of patient lists and reminder to do ACP, high priority patients flagged on EMR dashboard. | Salience | HS intervention | ACP, EMR, patients | N=341, 3% | N=910, 35% |
| 15 | Goldstein et al. 2019, US^41^ | RCT | IP and OP, No | EMR, e-mail/fax/texts/telephone | Physician plus other clinicians | Automated EMR reminders stating patient might benefit from GOCD and ICD deactivation. | Salience | Other clinician intervention | GOCD, EMR and patient survey, patients | N=222, 38.7% | N=301, 46.5% |
| 16 | Haley et al. 2017, US^16^ | Uncontrolled pre- and post- study | IP, No | EMR | Physicians only | Electronic text alerts sent as a reminder | Salience | Other clinician intervention | GOCD, EMR, patients | N=73, 20.5% | N=56, 44.6% |
| 17 | Halpert et al. 2022, US^17^ | Uncontrolled pre- and post- study | OP, No | EMR | Physicians only | EMR alert to consider ACP and reminder that patients were nudged to discuss ACP | Salience | Patient intervention | ACP, EMR, patients | N=426, 0% | N=426, 29.8% |
| 18 | Hanson et al. 2005, US^18^ | Uncontrolled pre- and post- study | IP, No | In-person/group meetings | Non-physician clinicians | Monthly action plans and performance data were sent, refreshments during educational sessions | Commitment, incentives, salience | Other clinician intervention | End-of-life care preferences, PMR, patients | N=345, 4% | N=346, 17% |
| 19 | Hanson et al. 2017, US^19^ | Uncontrolled pre- and post- study | IP, Yes | In-person/group meetings | Physicians only | Oncologists were contacted to consider palliative care consultation. | Salience | Other clinician and HS intervention | GOCD, EMR, patients | N=108, 29% | N=64, 48$ |
| 20 | Hayek et al. 2014, US^20^ | Uncontrolled pre- and post- study | OP, No | EMR, e-mail/fax/texts/telephone | Physicians only | E-mails were sent as a reminder to discuss AD, EMR visual reminder for high-risk patients | Salience | Patient intervention | AD, EMR, patients | N=100, 0% | N=588, 1.3% |
| 21 | Haynes et al. 2019, US^21^ | Uncontrolled pre- and post- study | IP, No | E-mail/fax/texts/telephone, in-person/group meetings | Physicians only | Automated e-mails identifying patients, monthly feedback e-mails about completion rates for each team, financial incentive (20$ in educational funds) | Incentives, norms, salience | Other clinician and HS intervention | POLST completion, EMR, patients | N=65, 38% | N=71, 51% |
| 22 | Heiman et al. 2004, US^42^ | RCT | OP, No | EMR, in-person/group meetings | Physicians only | EMR reminders to enter AD discussions in the EMR, reminders printed on patient summary sheets | Salience | Patient intervention | AD, EMR and PMR, patients | N=334, 2.7% | N=360, 13% |
| 23 | Horecki et al. 2024, US^22^ | Uncontrolled pre- and post- study | OP, Yes | EMR, in-person/group meetings | Physician plus other clinicians | EMR dashboard tracking of documentation, feedback shared during meetings to review the dashboards | Norms, salience | Other clinician and HS intervention | AD, EMR, patient records | N=2579, 35.1% | N=1286, 43.7% |
| 24 | Kantor et al. 2021, US^23^ | Uncontrolled pre- and post- study | IP, No | EMR, in-person/group meetings | Clinicians (not specified) | Alert to document code status, monthly feedback on ACP documentation, ACP documentation as a unit quality metric, mechanism for reimbursement to promote ACP code use | Incentives, salience, commitment | Other clinician and HS intervention | ACP, EMR, patient encounters | N=13143, 11.3% | N=16117, 24.6 |
| 25 | Karim et al. 2018, Canada^24^ | Uncontrolled pre- and post- study | OP, Yes | E-mail/fax/texts/telephone, in-person/group meetings | Physicians only | E-mails with patient list, visual reminders in patient’s paper record to discuss and document GOC, display in hallways GOC rates for each clinician | Norms, salience | Other clinician intervention | GOCD, EMR, aggregated monthly | N = not reported, Baseline: 0% | N = not reported, Active phase: 29% |
| 26 | Lakin et al. 2017, US^6^ | Non-randomized control study | IP and OP, No | E-mail/fax/texts/telephone, in-person/group meetings | Clinicians (not specified) | E-mails sent to coordinate SIC timing with clinicians. | Salience | Other clinician intervention | SIC, EMR, patients | N=77,3% | N=101,44.4% |
| 27 | Lee et al. 2022, US^43^ | RCT | IP, No | E-mail/fax/texts/telephone, in-person/group meetings | Clinicians (not specified) | Patient-specific form on GOC and preferences | Priming | Patient intervention | GOCD, EMR, patients | N=75, 8% | N=75, 21% |
| 28 | Levy et al. 2024, US^5^ | Non-randomized control study | OP, No | E-mail/fax/texts/telephone | Clinicians (not specified) | Feedback reports were e-mailed to clinicians which showed the number of templates completed | Salience | No other intervention | End-of-life preferences discussion, EMR, aggregated at intervention-site level | Not reported | Not reported |
| 29 | Lindner et al. 2007, US^25^ | Uncontrolled pre- and post- study | IP, No | EMR | Physician plus other clinicians | EMR alert to complete AD discussion | Salience | HS intervention | AD, EMR, patient admissions | N=117, 4% | N=107, 63% |
| 30 | Ma et al. 2020, Canada^8^ | Non-randomized control study | IP, No | E-mail/fax/texts/telephone, in-person/group meetings | Physician plus other clinicians | Unit champion reminded clinicians in person or through text to have an SIC | Salience | Other clinician, HS and patient intervention | SIC, EMR and PMR, patients | N=56, 50% | N=56, 98% |
| 31 | Manz et al. 2020, US^44^ | RCT | OP, Yes | E-mail/fax/texts/telephone | Physicians only | Weekly emails comparing SIC rates for all patients against peers’ rates and list of patients, opt-out reminder texts to conduct SICs | Norms, default, salience | No other intervention | SIC, EMR, patient encounters | N=12170, 1.3% | N=13889, 4.6% |
| 32 | Manz et al. 2023, US^45^ | RCT | OP, Yes | E-mail/fax/texts/telephone | Physicians only | Weekly emails comparing SIC rates against peers’ rates and list of patients, opt-out reminder texts to conduct SICs | Norms, default, salience | No other intervention | SIC, EMR, patient encounters | N=12356, 1.3% | N=28665, 4.4% |
| 33 | Mathew et al. 2023, US^26^ | Uncontrolled pre- and post- study | IP, No | EMR | Physician plus other clinicians | EMR reminder to complete ACP documentation, follow-up message was sent if ACP was not documented. | Salience | No other intervention | ACP, EMR, patients | N = not reported, 6 months pre-intervention: 15.3% | N = not reported, Intervention period: 21% |
| 34 | Morrison et al. 2005, US^46^ | RCT | IP, No | In-person/group meetings | Non-physician clinicians | Flagging of advance directives on patient charts, review of progress during team meetings | Salience | Other clinician intervention | ACP, PMR and patient survey, patients | N=96, 5% | N=43, 5% |
| 35 | Oppenheim et al. 2022, US^27^ | Uncontrolled pre- and post- study | OP, Yes | In-person/group meetings | Physicians only | Clinicians received a list of patients with no ACP and their ACP completion rate compared with their peers | Messenger, norms, salience | Patient intervention, HS intervention | ACP, EMR, aggregated monthly | N = not reported, baseline: 37% | N = not reported, end of implementation: 57% |
| 36 | Paladino et al. 2019, US^47^ | RCT | OP, Yes | E-mail/fax/texts/telephone | Physician plus other clinicians | E-mail reminders to initiate SIC | Salience | Other clinician, HS and patient intervention | SIC, EMR, patients | N=54, 11% | N=57, 61% |
| 37 | Patel et al. 2024, US^28^ | Uncontrolled pre- and post- study | IP, Yes | E-mail/fax/texts/telephone | Physician plus other clinicians | E-mails were sent to clinicians to initiate ACP conversations | Salience | HS intervention | ACP, EMR, patients | N=88, 2.3% | N=77, 80.5% |
| 38 | Pearlman et al. 2005, US^48^ | RCT | OP, No | Patient-held visual cues | Physician plus other clinicians | Patients flag and remind their clinicians to discuss ACP | Salience | Patient intervention | ACP, PMR, EMR and patient survey, patients | N=129, 38% | N=119, 64% |
| 39 | Picker et al. 2016, US^49^ | RCT | IP, No | In-person/group meetings | Physician plus other clinicians | Automated early warning system alert developed to identify patients at risk of clinical deterioration. | Priming | No other intervention | AD, EMR, patients | N=117, 15.4% | N=89, 37.1% |
| 40 | Pollak et al. 2019, US^50^ | RCT | IP, No | EMR | Physicians only | EMR notifications sent to clinicians to initiate GOC conversations with patients | Salience | Other clinician intervention | ACP, EMR, patients | N=159, 6% | N=269, 12% |
| 41 | Reed et al. 2020, US^29^ | Uncontrolled pre- and post- study | IP, No | EMR | Clinicians (not specified) | EMR reminders complete POLST, peer comparison feedback sent on POLST completion performance | Norms, salience | HS intervention | POLST completion, EMR, aggregated annually | N = not reported, in 2014: 41% | N = not reported, In 2019: 75% |
| 42 | Reinke et al. 2017, US^51^ | RCT | OP, No | In-person/group meetings | Clinicians (not specified) | 1-page patient-specific feedback form the form to intervention clinicians. | Priming | Patient intervention | End-of-life preferences discussion EMR, patients | N=81, 72% | N=76, 75% |
| 43 | Schell et al. 2023, US^30^ | Uncontrolled pre- and post- study | IP, No | EMR, e-mail/fax/texts/telephone | Clinicians (not specified) | EMR and e-mail alert sent to conduct GOC discussion with patients | Salience | Patient intervention, HS intervention | GOCD, EMR, patients | N=12571, 3.7% | N=10761, 9.3% |
| 44 | Serna et al. 2024, US^31^ | Uncontrolled pre- and post- study | IP, No | EMR, e-mail/fax/texts/telephone | Physician plus other clinicians | Weekly emails to consider SIC as flagged in the EMR dashboard | Salience | Patient intervention, HS intervention | SIC, EMR, patient encounters | N=2194, 3.5% | N=685, 2.8% |
| 45 | Sorge et al. 2024, US^32^ | Uncontrolled pre- and post- study | IP, No | EMR, e-mail/fax/texts/telephone | Physicians only | Code status EMR alert during patient stay, e-mail notification about the intervention | Salience | Other clinician intervention | Code status, EMR, patient charts | N=1828, 19.6% | N=1775, 31.4% |
| 46 | Switzer et al. 2019, US^33^ | Uncontrolled pre- and post- study | IP, Yes | EMR | Physicians only | EMR alert display to update code status documents | Salience | Other clinician intervention | Code status, EMR and PMR, patient encounters | N=244, 47.1% | N=291, 61.5% |
| 47 | Takvorian et al. 2024, US^52^ | RCT | OP, Yes | E-mail/fax/texts/telephone | Physician plus other clinicians | Text messages to complete SICs, weekly peer comparisons of SIC completion rates | Norms, salience | Patient intervention | SIC, EMR, patients | N=1004, 11.2% | N=1270, 14.1% |
| 48 | Temel et al. 2013, US^34^ | Uncontrolled pre- and post- study | OP, Yes | E-mail/fax/texts/telephone | Physician plus other clinicians | E-mail prompts to remind them to discuss GOC | Salience | No other intervention | Code status, EMR, patients | N=83, 14.5% | N=98, 33.7% |
| 49 | Topoll et al. 2022, US^35^ | Uncontrolled pre- and post- study | IP, No | E-mail/fax/texts/telephone, in-person/group meetings | Physician plus other clinicians | Monthly e-mails reminder to document code status | Salience | Other clinician intervention | Code status, EMR, aggregated monthly | N = not reported, Pre-intervention: 7.8% | N = not reported, 6-month follow-up: 45.2% |
| 50 | Wasp et al. 2022, US^36^ | Uncontrolled pre- and post- study | OP, Yes | E-mail/fax/texts/telephone, in-person/group meetings | Clinicians (not specified) | Reminder to perform SIC | Salience | Other clinician and HS intervention | SIC, EMR, patients | N=63, 0% | N=63, >70% |
| 51 | Wissow et al. 2004, US^7^ | Non-randomized control study | OP, No | In-person/group meetings, posters/stickers | Physician plus other clinicians | Visual cue reminders to conduct ACP, study team follows up with clinicians and provides feedback, food incentives to best performing clinics. | Incentives, salience | Patient and other clinician intervention | AD, EMR, patients | N=1277, 7.8% | N=843, 10.3% |

Notes: RCT: Randomized Control Trial, IP: Inpatient, OP: Outpatient, EMR: Electronic Medical Records, PMR: Paper Medical Records, SIC: Serious Illness Conversations, AD: Advance Directives, POLST/MOLST: Physician or medical order for life sustaining treatment, GOCD: Goals-of-care discussion, ACP: Advance care planning,

**Table A5. Studies assessing secondary outcomes**

|  | **Author/ Year/ Country** | **Study type** | **Nudge type** | **Secondary outcome** | **Palliative care consultations/referrals** | **Quality of communication** | **Hospital admission** | **Hospital length of stay** | **ICU admission** | **ICU length of stay** |
| --- | --- | --- | --- | --- | --- | --- | --- | --- | --- | --- |
| 1 | Bhattacharya et al. 2023^9^ | Quality improvement study | Salience | Healthcare utilization |  |  | positive |  |  |  |
| 2 | Brown et al. 2022^38^ | Randomized control trial | Salience | Palliative care referral/ consultation | negative |  |  |  |  |  |
| 3 | Chi et al. 2023^2^ | Quasi-experimental study | Salience | Healthcare utilization, palliative care referral/ consultation | positive |  | negative | negative |  |  |
| 4 | Conduit et al. 2021^12^ | Quality improvement study | Priming | Palliative care referral/ consultation | negative |  |  |  |  |  |
| 5 | Courtright et al. 2019^3^ | Quasi-experimental study | Default | Healthcare utilization, palliative care referral/ consultation | positive |  | negative | negative | negative | negative |
| 6 | Curtis et al. 2018^39^ | Randomized control trial | Priming | Quality of conversations |  | positive |  |  |  |  |
| 7 | Curtis et al. 2023^40^ | Randomized control trial | Priming, salience | palliative care referral/ consultation | negative |  |  |  |  |  |
| 8 | Deptola and Riggs 2019^13^ | Quality improvement study | Salience | Healthcare utilization, palliative care referral/ consultation | negative |  |  |  | positive |  |
| 9 | Fang et al. 2022^15^ | Quality improvement study | Salience | Palliative care referral/ consultation | negative |  |  |  |  |  |
| 10 | Goldstein et al. 2019^41^ | Randomized control trial | Salience | Palliative care referral/ consultation, other discussion | negative |  |  |  |  |  |
| 11 | Haley et al. 2017^16^ | Quality improvement study | Salience | Healthcare utilization, palliative care referral/ consultation | negative |  |  |  |  |  |
| 12 | Hanson et al. 2005^18^ | Quality improvement study | Salience, commitment, incentives | Palliative care referral/ consultation, patient outcomes | positive |  |  |  |  |  |
| 13 | Hanson et al. 2017^19^ | Quality improvement study | Salience | Healthcare utilization, palliative care referral/ consultation, patient outcomes | positive |  |  |  |  |  |
| 14 | Horecki et al. 2024^22^ | Quality improvement study | Salience, norms | Quality of conversations |  | positive |  |  |  |  |
| 15 | Karim et al. 2018^24^ | Quality improvement study | Salience, norms | Palliative care referral/ consultation | negative |  |  |  |  |  |
| 16 | Lakin et al. 2017^6^ | Non-randomized studies | Salience | Quality of conversations, palliative care referral/ consultation, timing of documentation | negative |  |  |  |  |  |
| 17 | Manz et al. 2023^45^ | Randomized control trial | Norms, salience, default | Healthcare utilization | negative |  |  |  | negative |  |
| 18 | Patel et al. 2024^28^ | Quality improvement study | Salience | Healthcare utilization | negative |  | negative | negative | negative | negative |
| 19 | Picker et al. 2016^49^ | Randomized control trial | Priming | Healthcare utilization, palliative care referral/ consultation | negative |  |  | negative | positive | positive |
| 20 | Pollak et al. 2019^50^ | Randomized control trial | Salience | Healthcare utilization, palliative care referral/ consultation | negative |  | positive | negative |  |  |
| 21 | Serna et al. 2024^31^ | Boston, Massachussets, United States | Salience | Healthcare utilization | positive |  |  |  | negative |  |
| 22 | Takvorian et al. 2024^52^ | Randomized control trial | Salience, norms | Healthcare utilization, palliative care referral/ consultation | negative |  |  |  |  |  |

**Positive** – significant increase in palliative care consultations/referrals; significant improvement in quality of communication; significant reduction in hospital length of stay and admissions/re-admissions, significant reduction in ICU admissions and ICU length of stay

**Negative** – non-significant or no difference in palliative care consultations/referrals; quality of communication; hospital length of stay and admissions/re-admissions, ICU admissions and ICU length of stay

**References**

1. McGuinness L, Higgins J. Risk-of-bias VISualization (robvis): An R package and Shiny web app for visualizing risk-of-bias assessments. *Res Syn Meth*. 2020;

2. Chi S, Kim S, Reuter M, et al. Advanced Care Planning for Hospitalized Patients Following Clinician Notification of Patient Mortality by a Machine Learning Algorithm. *JAMA Network Open*. 2023;6(4):e238795.

3. Courtright KR, Chivers C, Becker M, et al. Electronic Health Record Mortality Prediction Model for Targeted Palliative Care Among Hospitalized Medical Patients: a Pilot Quasi-experimental Study. *J Gen Intern Med*. 2019;34(9):1841-1847.

4. Gensheimer MF, Gupta D, Patel MI, et al. Use of Machine Learning and Lay Care Coaches to Increase Advance Care Planning Conversations for Patients With Metastatic Cancer. *JCO Oncology Practice*. 2022;19(2)

5. Levy C, Kononowech J, Ersek M, Phibbs CS, Scott W, Sales A. Evaluating feedback reports to support documentation of veterans’ care preferences in home based primary care. *BMC Geriatrics*. 2024;24(389)

6. Lakin JR, Koritsanszky LA, Cunningham R, et al. A Systematic Intervention To Improve Serious Illness Communication In Primary Care. *Health Aff (Millwood)*. Jul 1 2017;36(7):1258-1264. doi:10.1377/hlthaff.2017.0219

7. Wissow LS, Belote A, Kramer W, Compton-Phillips A, Kritzler R, Weiner JP. Promoting Advance Directives Among Elderly Primary Care Patients. *J Gen Intern Med*. 2004;19:944-951.

8. Ma C, Riehm LE, Bernacki R, Paladino J, You JJ. Quality of clinicians’ conversations with patients and families before and after implementation of the Serious Illness Care Program in a hospital setting: a retrospective chart review study. *CMAJ Open*. 2020;8(2)

9. Bhattacharya A, Chakrabartya S, Cabrales J, et al. Implementation of a palliative care consultation trigger tool for hospitalised patients with acute decompensated heart failure. *BMJ Open Quality*. 2023;12

10. Casarett D, Lakis K, Ma JE, et al. Goal-Concordant Care: End-of-Life Planning Conversations for All Seriously Ill Patients. *NEJM Catalyst*. 2022;3(12)

11. Colley A, Lin J, Pierce L, et al. Experiences with targeting inpatient advance care planning for emergency general surgery patients: A resident-led quality improvement project. *Surgery*. 2023;174:844-850.

12. Conduit C, Thompson M, Thomas R, Nott L, Wuttke M. Implementing 'Goals of Care' discussion and palliative care referral for patients with advanced lung cancer: an outpatient-based pilot project. *Intern Med J*. Apr 2021;51(4):540-547. doi:10.1111/imj.14817

13. Deptola AZ, Riggs J. Inpatient Goals-of-Care Conversations Reduce Intensive Care Unit Transfers in High-Risk Patients. *American Journal of Hospice & Palliative Medicine*. 2019;36(7):583-586.

14. Dutta PA, Flynn SJ, Oreper S, Kantor MA, Mourad M. Across race, ethnicity, and language: An intervention to improve advance care planning documentation unmasks health disparities. *J Hosp Med*. Jan 2024;19(1):5-12. doi:10.1002/jhm.13248

15. Fang YM, Peralta SSd. Triggering goals of care conversations in heart failure patients. *Journal of the American Association of Nurse Practitioners*. 2022;34(10)

16. Haley EM, Meisel D, Gitelman Y, Dingfield L, Casarett DJ, O’Connor NR. Electronic Goals of Care Alerts: An Innovative Strategy to Promote Primary Palliative Care. *Journal of Pain and Symptom Management*. 2017;53(5)

17. Halpert KD, Ward K, Sloane PD. Improving Advance Care Planning Documentation Using Reminders to Patients and Physicians: A Longitudinal Study in Primary Care. *American Journal of Hospice & Palliative Medicine*. 2022;39(1):62-67.

18. Hanson LC, Reynolds KS, Henderson M, Pickard CG. A Quality Improvement Intervention to Increase Palliative Care in Nursing Homes. *Journal of Palliative Medicine*. 2005;8(3)

19. Hanson LC, Collichio F, Bernard SA, et al. Integrating Palliative and Oncology Care for Patients with Advanced Cancer: A Quality Improvement Intervention. *Journal of Palliative Medicine*. 2017;20(12)

20. Hayek S, Nieva R, Corrigan F, et al. End-of-Life Care Planning: Improving Documentation of Advance Directives in the Outpatient Clinic Using Electronic Medical Records. *Journal of Palliative Medicine*. 2014;17(12)

21. Haynes CA, Dashiell-Earp CN, Wenger NS, et al. Improving Communication About Resuscitation Preference for Patients Discharged from Hospital to Nursing Home: A Quality Improvement Project. *Journal of Palliative Medicine*. 2019;22(5)

22. Horecki P, Deming J, Lagunas M, et al. Improve Advance Care Planning: A Brief Report Discussing Goals of Care Interventions to Improve Communication Among Health Care Teams and Patients Maximizing the Use of the Electronic Health Record Tools. *Journal of Palliative Medicine*. 2024;27(5)

23. Kantor MA, Scott BS, Abe-Jones Y, Raffel KE, Thombley R, Mourad M. Ask About What Matters: An Intervention to Improve Accessible Advance Care Planning Documentation. *Journal of Pain and Symptom Management*. 2021;62(5)

24. Karim S, Harle I, O’Donnell J, Li S, Booth CM. Documenting Goals of Care Among Patients With Advanced Cancer: Results of a Quality Improvement Initiative. *Journal of Oncology Practice*. 2018;14(9)

25. Lindner SA, Davoren JB, Vollmer A, Williams B, Landefeld CS. An Electronic Medical Record Intervention Increased Nursing Home Advance Directive Orders and Documentation. *Journal of the American Geriatrics Society*. 2007;55:1001-1006.

26. Mathew T, Patel A, DiGrande K, Michelis ND, Mody B, Lombardo D. Improving Advance Care Planning for Hospitalized Patients With Heart Failure. *Palliative Med Reports*. 2023;4

27. Oppenheim S, Figlin RA, Seferian EG, Reed M, Irwin SA, Rosen BT. Advance Care Planning in Patients With Metastatic Cancer: A Quality Improvement Initiative. *JCO Oncology Practice*. 2022;18(10)

28. Patel MN, Mara A, Acker Y, et al. Machine Learning for Targeted Advance Care Planning in Cancer Patients: A Quality Improvement Study. *Journal of Pain and Symptom Management*. 2024;68(6)

29. Reed MR, Stewart S, Meyer SA, Seferian EG, Harry C. Sax. Enhancing POLST Completion in a Hospital Setting: An Interdisciplinary Approach. *Journal of Healthcare Management*. 2020;65(6):397-405.

30. Schell JO, Schenker Y, Piscitello G, et al. Implementing a Serious Illness Risk Prediction Model: Impact on Goals of Care Documentation. *Journal of Pain and Symptom Management*. 2023;66(6)

31. Serna MK, Yoon C, Fiskio J, Lakin JR, Dalal AK, Schnipper JL. Using implementation science to encourage Serious Illness Conversations on general medicine inpatient services: An interrupted time series. *Journal of Hospital Medicine*. 2024:1-9.

32. Sorge J, Szpunar S, Daniel T, Saravolatz L. Using the Electronic Medical Record to Address Code Status Documentation: A Quality Improvement Project. *Journal of Healthcare Quality*. 2024;46(3):e1-e7.

33. Switzer B, Jazieh K, Bernstein E, Harris D. Impact of an Electronic Medical Record Alert on Code Status Documentation for Hospitalized Patients With Advanced Cancer. *JCO Oncology Practice*. 2019;16(3)

34. Temel JS, Greer JA, Gallagher ER, et al. Electronic Prompt to Improve Outpatient Code Status Documentation for Patients With Advanced Lung Cancer. *J Clin Oncol*. 2013;31(710-715)

35. Topoll AB, Wagner JK, Salem KM, Levenson JE, Makaroun MS, Arnold RM. Improving Code Status Documentation Rates Using Communication Skills Training in Vascular Surgery: A Quality Improvement Initiative. *Journal of Palliative Medicine*. 2022;25(4)

36. Wasp GT, Cullinan AM, Anton CP, et al. Interdisciplinary Approach and Patient/ Family Partners to Improve Serious Illness Conversations in Outpatient Oncology. *JCO Oncology Practice*. 2022;18(10)

37. Au DH, Udris EM, Engelberg RA, et al. A Randomized Trial to Improve Communication About End-of-Life Care Among Patients With COPD. *Chest*. 2012;141(3):725-735.

38. Brown C, Lee XJ, Farrington A, et al. Impact of a prospective feedback loop on care review activities in older patients at the end of life. A stepped‑wedge randomised trial. *BMC Geriatrics*. 2022;22(860)

39. Curtis JR, Downey L, Back AL, et al. Effect of a Patient and Clinician Communication-Priming Intervention on Patient-Reported Goals-of-Care Discussions Between Patients With Serious Illness and Clinicians: A Randomized Clinical Trial. *JAMA Internal Medicine*. 2018;178(7):930-940.

40. Curtis JR, Lee RY, Brumback LC, et al. Intervention to Promote Communication About Goals of Care for Hospitalized Patients With Serious Illness: A Randomized Clinical Trial. *JAMA*. Jun 20 2023;329(23):2028-2037. doi:10.1001/jama.2023.8812

41. Goldstein NE, Mather H, McKendrick K, et al. Improving Communication in Heart Failure Patient Care. *J Am Coll Cardiol*. 2019;74(13):1682-1692.

42. Heiman H, Bates DW, Fairchild D, Shaykevich S, Lehmann LS. Improving Completion of Advance Directives in the Primary Care Setting: A Randomized Controlled Trial. *Am J Med*. 2004;117(5):318-324.

43. Lee RY, Kross EK, Downey L, et al. Efficacy of a Communication-Priming Intervention on Documented Goals-of-Care Discussions in Hospitalized Patients With Serious Illness A Randomized Clinical Trial. *JAMA Network Open*. 2022;5(4):e225088.

44. Manz CR, Parikh RB, Small DS, et al. Effect of Integrating Machine Learning Mortality Estimates With Behavioral Nudges to Clinicians on Serious Illness Conversations Among Patients With Cancer: A Stepped-Wedge Cluster Randomized Clinical Trial. *JAMA Oncology*. 2020;6(12)

45. Manz CR, Zhang Y, Chen K, et al. Long-term Effect of Machine Learning-Triggered Behavioral Nudges on Serious Illness Conversations and End-of-Life Outcomes Among Patients With Cancer: A Randomized Clinical Trial. *JAMA Oncol*. Mar 1 2023;9(3):414-418. doi:10.1001/jamaoncol.2022.6303

46. Morrison RS, Chichin E, Carter J, Burack O, Lantz M, Meier DE. The Effect of a Social Work Intervention to Enhance Advance Care Planning Documentation in the Nursing Home. *Journal of the American Geriatrics Society*. 2005;53:290-294.

47. Paladino J, Bernacki R, Neville BA, et al. Evaluating an Intervention to Improve Communication Between Oncology Clinicians and Patients With Life-Limiting Cancer: A Cluster Randomized Clinical Trial of the Serious Illness Care Program. *JAMA Oncology*. 2019;5(6):801-809.

48. Pearlman RA, Starks H, Cain KC, Cole WG. Improvements in Advance Care Planning in the Veterans Affairs System: Results of a Multifaceted Intervention. *Arch Intern Med*. 2005;165:667-674.

49. Picker D, Dans M, Heard K, et al. A Randomized Trial of Palliative Care Discussions Linked to an Automated Early Warning System Alert. *Critical Care Medicine*. 2016;45(2)

50. Pollak KI, Gao X, Beliveau J, Griffith B, Kennedy D, Casarett D. Pilot Study to Improve Goals of Care Conversations Among Hospitalists. *J Pain Symptom Manage*. Nov 2019;58(5):864-870. doi:10.1016/j.jpainsymman.2019.06.007

51. Reinke LF, Feemster LC, McDowell J, et al. The long term impact of an end-of-life communication intervention among veterans with COPD. *Heart & Lung*. 2017;46(30-34)

52. Takvorian SU, Gabriel P, Wileyto EP, et al. Clinician- and Patient-Directed Communication Strategies for Patients With Cancer at High Mortality Risk A Cluster Randomized Trial. *JAMA Network Open*. 2024;7(7)
